# HIV and SARS-Coronavirus-2 Epidemics: Possible Interactions and Need for Studies, Especially in Africa

**DOI:** 10.3389/fmed.2020.00216

**Published:** 2020-05-12

**Authors:** Francesca Cainelli, Bartholomew Dzudzor, Massimiliano Lanzafame, Adonis Goushchi, Sirika Chhem, Sandro Vento

**Affiliations:** ^1^Raffles Medical Group Clinic, Phnom Penh, Cambodia; ^2^Faculty of Medicine, University of Puthisastra, Phnom Penh, Cambodia; ^3^Department of Medical Biochemistry, Korle-Bu, College of Health Sciences, University of Ghana Medical School, Accra, Ghana; ^4^Diagnosis and Treatment of HIV Infection Unit, University Hospital, Verona, Italy; ^5^Infectious Diseases, Hopital Erasme, Université Libre de Bruxelles, Brussels, Belgium; ^6^Faculty of Pharmacy, University of Puthisastra, Phnom Penh, Cambodia

**Keywords:** HIV, SARS-CoV-2, COVID-19, antiretrovirals (ARVs), Africa

## Introduction

The HIV pandemic characterized the end of the second millennium and spread all over the world. The SARS-Coronavirus-2 (SARS-CoV-2) pandemic is the most striking of the beginning of the third millennium, and is of particular concern especially for Africa, where most HIV-infected people live. As of 28 April 2020, all but three [Western Sahara, Comoros, and Lesotho) African countries were affected, with 33,566 COVID-19 cases, and 1,469 deaths (1). Africa has a young population (the median age of the 1.3 billion people is 19.7 years] (2) and this could diminish the severity of COVID-19 but also increase the number of asymptomatic subjects, leading to a wider, and difficult to detect epidemic (3). What are the implications of the SARS-CoV-2 pandemic for HIV-infected people, particularly in a continent where, in 2018, 25.7 million people lived with HIV, and 9.4 million were not on antiretrovirals (ARVs) (4)?

## Recent Studies

Even though a few, most recently published papers have dealt with aspects of the SARS-CoV-2 pandemic that may particularly affect people living with HIV, there are extremely few data in the literature on HIV-SARS-CoV-2 coinfections.

A number of published manuscripts have examined aspects other than the course of SARS-CoV-2 coinfection in HIV-infected individuals. In particular, the following have been discussed: SARS-CoV-2 coinfection as a further burden to people living with HIV, that may suffer from substance abuse, chronic non-communicable diseases, mental health issues, and other infections (5); the effects of the SARS-CoV-2 epidemic on HIV care and the stress related to the pandemic and to social distancing in HIV-infected people (6); the fact that COVID-19 is reducing the capacity of the United States health system to address effectively HIV prevention and care, and its associated endemic sexually transmitted infections (7); the impact of the SARS-CoV-2 pandemic in the area with the highest number of new HIV diagnoses in the United States (8); lessons learnt from dealing with the HIV pandemic which might help to tackle the SARS-CoV-2 pandemic (9).

Overall, few cases of SARS-CoV-2-HIV coinfections have been reported in the literature as of 28 April 2020. A survey done in patients in Wuhan reported no higher rates of COVID-19 in HIV-infected vs. non-HIV-infected people, and no increased risk with low CD4 cell count (10). All eight patients with CT scan compatible with COVID-19 had undetectable HIV-RNA at the last assessment (within 3 months), six had positive SARS-CoV-2 swabs, two had CD4 cell count below 350/μL at the last assessment. One HIV-coinfected patient died, and another had a severe COVID-19 (10). An additional, SARS-CoV-2 infected but asymptomatic HIV-coinfected patient had a very low CD4 cell count (27/μl), was treated with chemotherapy for Kaposi's sarcoma, and had been on ARVs for only 1 month (10).

A 24-year-old, Chinese HIV-infected patient with a 2-year treatment history with tenofovir, lamivudine and efavirenz (CD4 cell count and HIV-RNA levels unreported), had a non-severe course of COVID-19 (11). Lopinavir/ritonavir had been added to the antiretroviral regimen after COVID-19 diagnosis (11).

A further Chinese patient living with HIV had 34 CD4 cells/μL and a prolonged course of COVID-19 (12). An additional HIV-infected patient with fever, muscle aches and right lower lobe pneumonia at a chest CT scan was reported by Chinese authors from Shenzhen (13). However, SARS-CoV-2 RNA was persistently negative on different specimen samples at various times during the course of his illness (13), and we cannot therefore be sure that this patient was SARS-CoV-2-coinfected.

A 66-year-old American man living with HIV and with undetectable HIV-RNA died of COVID-19 pneumonia (14).

Five HIV-coinfected patients have been reported from Spain (15). Four patients were on ARVs, and had CD4 cell counts higher than 400/μL and undetectable HIV-RNA; one patient was ARV-naïve, had 13 CD4 cells/μL and HIV-RNA 45,500 copies/mL. Two patients were admitted to intensive care (one of them being the ARV-naïve patient), four had been discharged, and one (with CD4 cell count >400/μL) remained in intensive care at the time of submission of the manuscript (15). Three patients were treated with lopinavir/ritonavir and two were given darunavir/cobicistat.

Three HIV-coinfected cases have been reported from Italy (16). A 62-year-old man with undetectable viral load and 441 CD4 cells/μL required mechanical ventilation and improved; a 63-year-old man with undetectable HIV-RNA and 743 CD4 cells/μL and a 57-year-old woman (HIV-RNA and CD4 cell count not reported) had an uneventful course (16). Interestingly, prior to getting SARS-CoV-2 all the three patients were on darunavir-based antiretroviral therapy, and pharmacokinetic data showed good compliance, suggesting that darunavir, at least at the currently employed 800 mg dosage, does not prevent SARS-CoV-2 infection HIV-infected individuals (16). It must be stressed that Janssen reported on March 18, 2020, that darunavir is not effective against SARS-CoV-2 due to low affinity to coronavirus protease.

## Discussion

It is impossible to draw conclusions from the extremely small number of SARS-CoV-2-HIV-coinfected patients reported in the literature as of 28 April 2020. However, there are a number of possible interactions between HIV and SARS-CoV-2 that needs to be clarified in large studies.

Patients on antiretrovirals and with CD4 cell counts higher than 200/μL might have a mild or moderate course of COVID-19, should ARVs have an effect on SARS-CoV-2. Protease inhibitors, in particular, inhibit enzymes which activate envelope glycoproteins as part of the process of viral entry into cells (17). Even though lopinavir/ritonavir treatment was of no particular benefit in a randomized, controlled, open-label trial in hospitalized adult Chinese patients with severe COVID-19 (18), this drug combination given at an earlier stage of disease might be beneficial. Perhaps antiretrovirals might also help to prevent SARS-CoV-2 infection, as suggested for SARS-1 (19) and MERS (20). However, on the basis of available evidence, a recently published review concluded that it is unclear whether lopinavir/ritonavir and other ARVs improve clinical outcomes in severe COVID-19 or prevent SARS-CoV-2 infection in patients at high risk of acquiring it (21).

An issue of particular concern is that a high number of respiratory viral infections (with a considerable severity requiring ICU care), including other coronavirus infections, have been found not only in HIV-infected patients with low CD4 cell count and high viral load but also in individuals with undetectable HIV-RNA (22). In urban South Africa, the death rate for influenza-associated severe acute respiratory illness is 20-fold higher in HIV-infected than in uninfected subjects (23). On the basis of these data, COVID-19 might be more severe and determine a higher death rate in HIV-infected patients.

Preliminary data from China indicate that patients with moderate or severe COVID-19 have reduced or very reduced (<200/μL) numbers of CD4 cells (24). Theoretically, this would put untreated HIV-infected patients with low CD4 cell numbers at particularly high risk of superimposed opportunistic infections during COVID-19. They might also be more susceptible to SARS-CoV-2 acquisition. Higher serum levels of pro-inflammatory cytokines, including IL-6, are found in severe cases of COVID-19, and thought to contribute to a fatal outcome (25). Elevated IL-6 levels are associated with older age, higher body mass index, higher viral replication and low nadir CD4+ cell count in HIV-infected patients, and predicts poor CD4 cell recovery in subjects starting ARVs (26). Whether pre-existing elevated levels of IL-6 will lead to a worse outcome for HIV-SARS-CoV-2 coinfected patients remains to be established.

Tuberculosis coinfection is a huge problem in people living with HIV. In particular, in 2016, 2.5 million new cases of TB occurred in Africa, and an estimated 417,000 people died from the disease (over 25% of TB deaths worldwide) (27). In South Africa, tuberculosis coinfection is associated with greater mortality in subjects with influenza, and influenza coinfection is associated with higher mortality in people with tuberculosis (28). Could it be the same for TB-COVID-19? Chronic lung damage secondary to tuberculosis might also play a role in COVID-19 negative outcome. In the case of influenza, studies in mice showed that the amount of tissue damage among tuberculosis–influenza-coinfected mice increased with longer duration of tuberculosis before the challenge with influenza (29). Hence, a serious illness might develop during a SARS-CoV-2 infection not only in patients with TB but also in those with pulmonary TB history. Regulatory T cell numbers increase (30) and CD4 cells decrease (31) in patients with TB; interestingly, during the 2003 SARS epidemic, TB-SARS coinfection led to more striking CD4 cell decreases and poorer anti-SARS IgG antibody responses in the few patients studied (32). Whether TB leads to a similar impairment in the immune response to SARS-CoV-2 needs to be established.

In addition, in countries or areas with high TB burden, it won't be easy to distinguish between TB and COVID-19, as symptoms may be similar; in this respect, it will be very important to collect a proper clinical history that will allow to distinguish one from the other. Unfortunately, this will be difficult, should COVID-19 cases increase considerably. A further, concerning issue is that people with possible tuberculosis may avoid to seek hospital treatment for fear to get SARS-CoV-2 infection, as happened during the 2003 SARS epidemic (33).

The co-existence of the two epidemics of HIV and SARS-CoV-2 could be particularly deleterious for people living with HIV not only in low and middle-income countries but also in high income countries. Widespread lockdowns, enforced in an attempt to curb the spread of SARS-CoV-2 infection, lead to patients' job losses, difficulties in reaching the clinics where anti-HIV drugs are distributed, and problems in drug supplies to the clinics. Funds needed to step up the response to the new pandemic could reduce those assigned to the fight against HIV infection/AIDS and TB, and vulnerable HIV-infected populations (drug users, sex workers, poor patients living in urban slums or in rural areas, prisoners) would particularly suffer from this. Any efforts will have to be made to prevent or limit the above problems. In any case, it will be extremely important to describe the features of SARS-CoV-2 infection and the evolution of COVID-19 in HIV-infected patients, including whether HIV-infected people develop sufficient level of anti-SARS-CoV-2 antibodies, and their persistence over time. Even in this difficult situation, clinical and research centers, including those in Africa, will have to strive to clarify the numerous aspects of this unprecedented coinfection for the benefit of all HIV-infected patients worldwide.

## Author Contributions

FC and SV had the idea of writing the manuscript. FC drafted the manuscript with help from BD and ML. AG and SC contributed to literature search. SV revised the first manuscript draft. All authors revised the second draft and approved the final version.

## Conflict of Interest

The authors declare that the research was conducted in the absence of any commercial or financial relationships that could be construed as a potential conflict of interest.
